# A Sensor-Aware Decoupled Learning Framework for Robust Multi-Scale Time-Series Forecasting in Oil Production Systems

**DOI:** 10.3390/s26113332

**Published:** 2026-05-24

**Authors:** Guojian Cheng, Wenhan Zhang, Zhonghui Jin, Lei Cai

**Affiliations:** 1 School of Computer Science, Xi’an Shiyou University, Xi’an 710065, China; gjcheng@xsyu.edu.cn; 2 School of Electronic Information, Xijing University, Xi’an 710123, China; 3 School of Software, Northwestern Polytechnical University, Xi’an 710129, China; jinzhonghui@mail.nwpu.edu.cn; 4 School of Computer Science and Engineering, Xi’an University of Technology, Xi’an 710048, China; cailei@xaut.edu.cn

**Keywords:** sensor-based forecasting, oil production monitoring, deep learning, temporal convolutional network, LightGBM, residual learning

## Abstract

Accurate forecasting of oil well production via field monitoring systems is significantly restricted by a structural conflict in modeling, where temporal dependency learning and nonlinear feature representation are closely coupled. Such coupling forces a trade-off between capturing long-term temporal dependencies and retaining sensitivity to short-term sensor fluctuations, while amplified local sensitivity easily increases noise interference and weakens model robustness under complex non-stationary sensor dynamics. To solve this problem, this study proposes a novel sensor-driven hybrid framework named Temporal Augmented Residual Network (TAR-Net), which adopts a decoupled paradigm to separate global temporal modeling and local fluctuation compensation explicitly. A multi-scale dilated Temporal Convolutional Network (TCN) extracts long-range temporal patterns from multi-source sensor data, and a LightGBM-based residual module conducts targeted error correction. Meanwhile, multi-scale temporal features and adaptive multi-fidelity Bayesian optimization are applied to enhance model adaptability. Validated on real sensor data from the Volve oilfield, TAR-Net surpasses 13 benchmark models with an R^2^ of 0.9832 and a MAPE of 7.8%. Residual and trajectory analyses verify its balance between global trend consistency and local fluctuation sensitivity. This framework offers a robust sensor-aware solution for complex multi-scale temporal modeling in industrial production systems.

## 1. Introduction

Oil production forecasting serves as a fundamental basis for dynamic analysis and production decision-making throughout the lifecycle of reservoir development, with its accuracy directly influencing development strategy optimization and economic evaluation [1]. With the rapid advancement of field monitoring systems, modern oil production increasingly relies on large-scale sensor networks that continuously capture critical operational variables. However, due to the expansion of unconventional resource exploitation, reservoir heterogeneity and multiphase flow complexity have intensified. Coupled with frequent operational adjustments, production systems are subject to persistent multi-source disturbances, resulting in sensor-derived time series that exhibit strong non-stationarity, nonlinear coupling, and multi-scale temporal dynamics. These dynamics typically manifest as long-term trends driven by reservoir depletion, medium-term oscillations induced by injection–production strategies, and short-term abrupt perturbations caused by operational disturbances or equipment anomalies. Under such complex conditions, developing a robust and adaptive forecasting framework capable of explicitly modeling heterogeneous temporal dynamics remains a critical yet challenging task.

To address these challenges, existing production forecasting approaches have evolved along three primary paradigms: empirical models, mechanism-driven models, and data-driven intelligent models. Empirical models rely on statistical fitting and lack the capacity to capture complex dynamic evolution. Mechanism-driven models attempt to describe reservoir behavior based on physical principles; however, they often suffer from strong parameter sensitivity and high computational cost, and their predictive reliability deteriorates significantly under frequent operational interventions and evolving production regimes [2]. In contrast, data-driven intelligent models have become dominant due to their superior nonlinear representation capability and flexibility in handling high-dimensional data [3].

From a signal-processing standpoint, the core challenge lies in separating these heterogeneous temporal components. Traditional decomposition methods (e.g., STL, wavelet transform) assume linear superposition and fixed basis functions, which cannot capture the nonlinear coupling and time-varying interactions present in production data. A learnable, adaptive representation is therefore required. Traditional decomposition methods, such as STL and wavelet transform, rely on linear superposition assumptions and predefined basis functions, which are inherently insufficient for capturing nonlinear coupling and time-varying interactions. Consequently, a learnable and adaptive representation mechanism is required to replace static decomposition paradigms.

From a modeling perspective, early data-driven approaches primarily relied on recurrent neural networks (RNNs), including LSTM and GRU, which model temporal dependencies through recursive state transitions [4,5]. While effective for short-term dependency modeling, their sequential nature leads to gradient degradation over long horizons and limits parallelization. To address these limitations, convolution-based and attention-based architectures have been extensively explored. Temporal convolutional networks (TCN) employ dilated causal convolutions to enlarge the receptive field and improve long-range dependency modeling [6], whereas Transformer-based models leverage self-attention mechanisms to capture global temporal correlations [7]. Nevertheless, these architectures predominantly enhance global structure modeling while exhibiting limited sensitivity to localized abrupt variations.

To further improve predictive performance, recent studies have investigated hybrid architectures, ensemble strategies, and external optimization techniques. Hybrid models, such as convolution–recurrent combinations and attention-augmented networks, enhance feature extraction through structural integration [8,9,10]. Tree-based ensemble methods, particularly LightGBM, provide strong nonlinear approximation capability with efficient training [11]. Additionally, hybrid frameworks combining statistical learning and deep models (e.g., random forest–LSTM) improve robustness under complex conditions [12], while bidirectional architectures further enhance temporal representation [13]. Meanwhile, optimization strategies such as Bayesian optimization, multi-fidelity optimization, and ensemble tuning have been introduced to alleviate hyperparameter sensitivity and improve generalization [14,15,16,17,18,19].

Despite these advancements, a fundamental limitation remains insufficiently addressed. The problem is compounded by varying sensor quality and noise across multi-source measurements. Most existing methods implicitly combine temporal dependency learning with nonlinear feature representation, never truly disentangling them. Consequently, model capacity is capped by architectural constraints, and post hoc optimization cannot overcome fundamental representational limits [20]. Distribution shifts across wells further worsen generalization [21].

More fundamentally, unified forecasting frameworks face an inherent structural conflict. Modeling global temporal dependencies favors smooth, consistent trends, whereas capturing nonlinear features demands high sensitivity to local irregularities. Under a single loss (e.g., MSE), the model is forced to minimize discrepancies across all scales simultaneously. This creates conflicting gradients: fitting long-term trends suppresses short-term variability, while chasing local fluctuations distorts the overall structure. The result is a compromise that is suboptimal for both objectives.

This trade-off appears across architectures—attention mechanisms that reweight features without altering the learning paradigm [22], Transformers that react slowly to local changes [23], tree-based models lacking explicit temporal dynamics, and knowledge-enhanced methods constrained by static representations [24]. Thus, whenever global trends and local perturbations coexist, unified models inevitably suffer from either over-smoothing or over-sensitivity [25,26,27,28].

To address this structural limitation, this study proposes a Temporal Augmented Residual Network (TAR-Net), which introduces an explicitly decoupled modeling framework for oil production forecasting. The key contributions of this work are summarized as follows: (1) A Novel Decoupled Learning Paradigm: We propose a framework that mathematically separates global temporal dependency modeling from local nonlinear fluctuation compensation. This explicit disentanglement fundamentally mitigates the inherent multi-objective conflict found in unified models, avoiding the trade-off between structural over-smoothing and local over sensitivity. (2) A Multi-Scale Hybrid Architecture: We design a targeted architecture where a multi-scale dilated temporal convolutional network (TCN) captures long-range temporal structures efficiently, while a LightGBM-based residual learning module is introduced to perform localized, nonlinear error correction by leveraging its sensitivity to local irregularities. (3) Enhanced Representation and Adaptive Optimization: We construct a multi-level feature representation strategy integrating rolling statistical descriptors and differencing-based dynamic features to enhance multi-scale perception. Furthermore, an adaptive multi-fidelity Bayesian optimization scheme is incorporated to significantly improve overall model robustness and generalization under heterogeneous production conditions.

## 2. Materials and Methods

### 2.1. Data Description

This study employs the publicly available Volve oilfield production dataset and selects time-series production data from three representative wells, F-11H, F-12H, and F-14H, as the primary research objects. To ensure data quality, a three-step cleaning procedure was applied. First, records with missing sensor readings were excluded. Second, physically implausible values (e.g., negative pressures) were removed based on operational constraints. Third, a 3σ statistical filter was employed: for each continuous variable, data points deviating from the mean by more than three standard deviations were identified as outliers and discarded. After cleaning, a total of 4662 valid samples from the three wells were retained. The dataset encompasses a comprehensive set of core variables, including the temporal index (DATEPRD), well identifier (WELL_BORE_CODE), average downhole pressure (AVG_DOWNHOLE_PRESSURE), average downhole temperature (AVG_DOWNHOLE_TEMPERATURE), average tubing pressure differential (AVG_DP_TUBING), average choke size (AVG_CHOKE_SIZE_P), average wellhead pressure (AVG_WHT_P), choke pressure differential (DP_CHOKE_SIZE), and the target variable, daily oil production (BORE_OIL_VOL), thereby providing a robust foundation for time-series forecasting of petroleum production. To preserve temporal causality and avoid data leakage, a chronological splitting strategy is adopted, whereby the earliest 80% of observations for each well are allocated to the training set, and the remaining 20% are reserved for testing. The detailed distribution of training and testing samples for each well is presented in Table 1.

### 2.2. Feature Engineering

In the task of oil well production forecasting, the input data are typically acquired from field monitoring systems, consisting of a limited set of sensor-derived observational variables. However, relying solely on these raw sensor measurements is insufficient to fully capture the complex temporal dynamics inherent in the production process. To enhance the model’s capability in representing multi-scale temporal structures, this study develops a systematic feature engineering strategy that extracts both statistical characteristics and dynamic variation patterns from sensor data across multiple temporal scales. This design facilitates effective feature-level support for the subsequent decoupled learning framework, enabling improved sensitivity to heterogeneous temporal behaviors.

From a sensor signal processing perspective, the constructed features can be interpreted as multi-scale temporal filters. Rolling statistical features approximate low-pass filtering behavior, capturing slow-varying trends in sensor measurements, while differencing operations emphasize high-frequency components associated with rapid fluctuations. These features are designed to capture the local mean level and the stability of fluctuations. Specifically, short-term windows primarily describe recent production states, whereas longer windows quantify the extent of production variability, thereby providing the model with production information across different temporal scales.

In contrast to static statistical descriptors, differential features are capable of capturing the dynamic behavior of production variations. The first-order difference represents the change in production between consecutive time steps, formulated as:

(1) Δy(t)=y(t)−y(t−1)

This feature characterizes the instantaneous rate of change in production, thereby reflecting short-term fluctuations and abrupt variations. Furthermore, the second-order difference describes the variation in the rate of change, given by:

(2) $$\Delta^{2}y\left(t\right)=y(t)-2y(t-1)+y(t-2)$$

When $\Delta^{2}y\left(t\right)$ > 0, it indicates an acceleration in production change; conversely, when $\Delta^{2}y\left(t\right)$ < 0, it suggests a deceleration in the rate of change.

**Figure 1:**
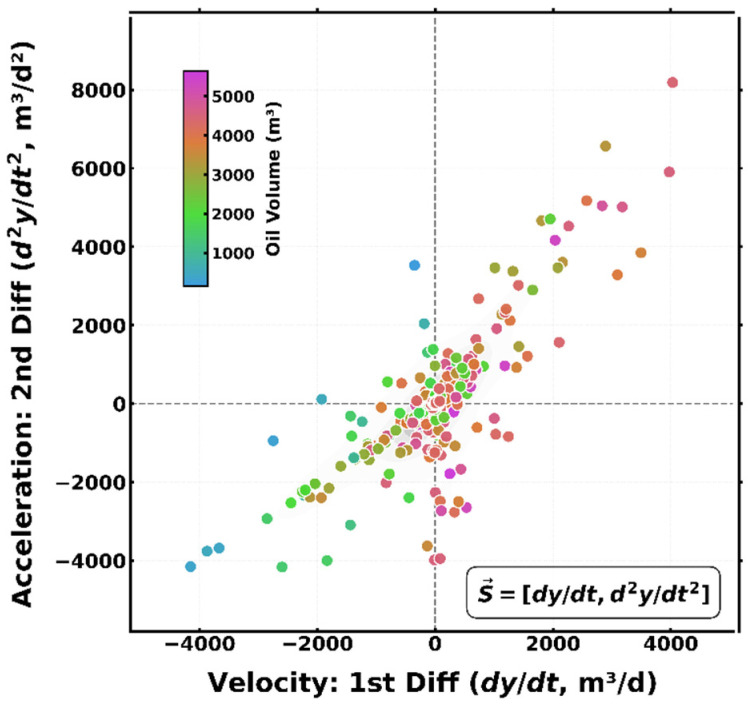
Phase space distribution of dynamic production characteristics for well F-14H.

To further investigate the dynamical structure embedded in the differential features, Figure 1 presents the phase-space scatter distribution formed by the first- and second-order differences of production from a representative oil well, F-14H. It can be observed that the majority of data points are concentrated around the origin, indicating that production variations remain relatively small during most operational stages, and the overall production state is comparatively stable. Meanwhile, the scatter points exhibit a band-like distribution along the diagonal direction, suggesting a certain degree of correlation between the rate of change and its acceleration; that is, when production increases or decreases, the process is often accompanied by corresponding acceleration or deceleration characteristics. In addition, data points corresponding to high-production stages are predominantly distributed on the right-hand side of the plot, whereas those associated with low-production stages are more frequently located on the left-hand side, reflecting pronounced differences in dynamic behaviors across distinct production phases.

**Figure 2:**
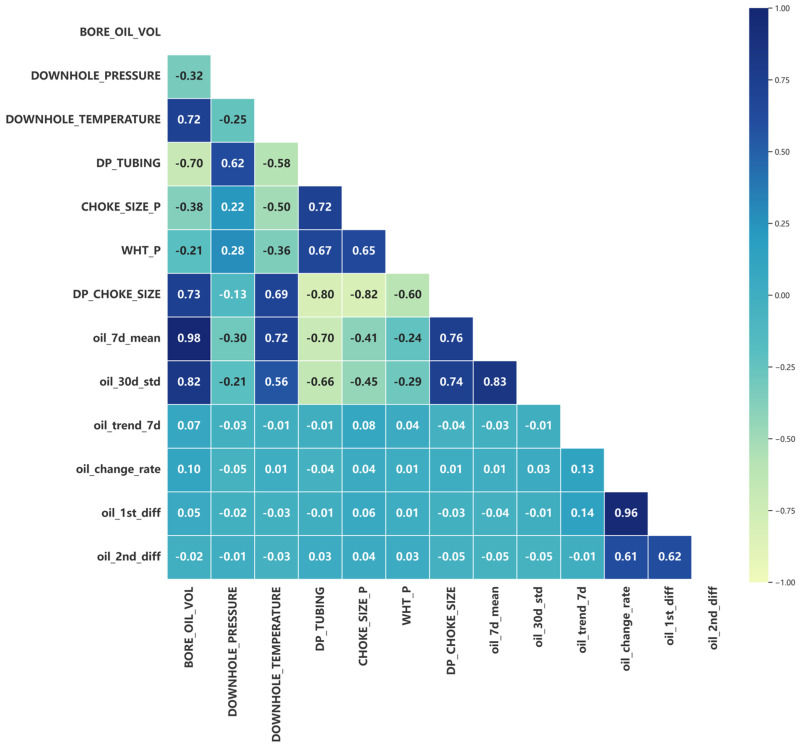
Correlation heatmap of features with bore oil volume.

To rigorously evaluate the effectiveness of the engineered features and to elucidate potential interdependencies among variables, we performed a systematic correlation analysis between all input features and the target variable. Pearson correlation coefficients were employed to quantify the degree of linear association between individual features and daily oil production, and a correlation heatmap was generated as shown in Figure 2 to visually delineate the relational architecture among the features [29]. The results reveal that statistical features such as rolling averages exhibit pronounced positive correlations with daily oil production, thereby effectively encapsulating the overall production levels and stability states of the oil wells; in contrast, dynamic features such as first- and second-order differencing display comparatively modest correlations with the raw production yet faithfully capture the transient fluctuation signatures embedded within the production evolution process. These findings underscore that differencing features primarily encode short-term dynamic variations, whereas rolling statistical features reflect longer-term production levels, with the two feature classes demonstrating robust complementarity in their representational capacities.

Based on the foregoing feature analysis, the present study ultimately incorporates both rolling statistical features and differencing-based dynamic attributes as joint model inputs, thereby furnishing a comprehensive multi-scale temporal representation of oil-well production yields. The statistical features delineate the overarching production state in terms of mean level and stability, whereas the differencing features capture the dynamic trajectories and fluctuation signatures embedded within the evolution of output. It should be emphasized that this explicit feature construction is not redundant with respect to the deep temporal backbone; rather, it serves to orthogonalize the input space, ensuring that the tree-based residual module—which lacks native temporal perception—receives an inductive bias specifically tailored to nonlinear fluctuation compensation. By simultaneously integrating these complementary feature families, the model preserves essential baseline production information while adeptly discerning short-term transients, thereby substantially augmenting its capacity to model the intricate dynamics of complex production processes.

### 2.3. Architecture of the TAR-Net Model

**Figure 3:**
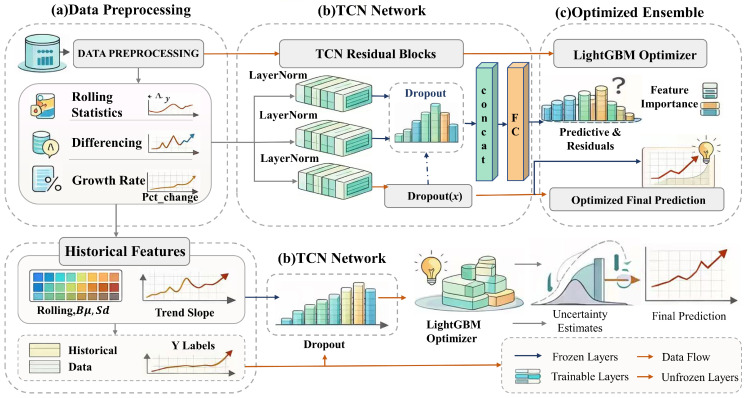
Overall architecture of the TAR-Net model.

To address the inherent structural conflict between global trend modeling and local fluctuation sensitivity observed in sensor-based time-series forecasting [30], the proposed Temporal Augmented Residual Network (TAR-Net) adopts a hierarchical and decoupled architecture. The design follows a strict division of responsibilities: a deep temporal backbone captures the continuous, low-frequency evolution of the production signal, while a gradient-boosting residual module compensates for the discontinuous, high-frequency perturbations that the backbone intentionally disregards. As illustrated in Figure 3, TAR-Net operates within a unified data encoding space wherein the raw time series, first- and second-order differences, and engineered statistical features are jointly projected into a common representation—with the crucial distinction that these feature groups will be routed preferentially toward the component for which they provide the most relevant inductive bias.

From an architectural perspective, TAR-Net is specifically designed to capture the multi-scale variability and localized instability inherent in oil well production sequences. The multi-scale dilated convolutions are employed to extract dynamic dependencies across diverse temporal horizons, whereas the residual learning module is dedicated to compensating for nonlinear discrepancies that are not readily captured by deep neural networks. These two components operate synergistically on trend modeling and error correction, respectively, thereby enabling the model to maintain robust and stable responses across different dynamic regimes.

From a modeling perspective, TAR-Net can be interpreted as a two-stage approximation framework, where the TCN captures the dominant temporal structure, and the residual module refines high-frequency components that are otherwise difficult to model using convolutional operations alone.

#### 2.3.1. TCN for Time Series Modeling

**Figure 4:**
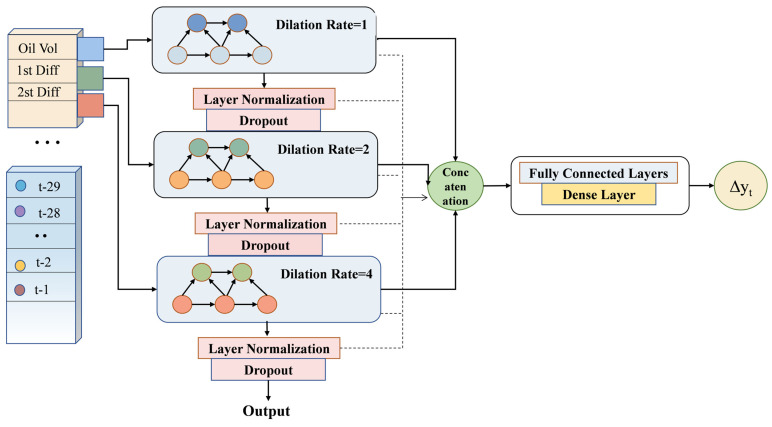
Detailed architecture of the multi-scale dilated TCN backbone.

The oil production process is inherently governed by a multitude of factors, including variations in reservoir pressure, the progressive increase in water cut, and adjustments in production schemes, all of which collectively induce pronounced non-stationarity and stage-wise dynamics in sensor-derived production time series. Within the proposed sensor-driven decoupled learning framework, accurately capturing global temporal dependencies is essential for modeling the long-term evolution of production dynamics. To this end, a temporal modeling module based on the Temporal Convolutional Network (TCN) is developed to effectively extract long-range temporal dependencies from multi-scale sensor data streams. The overall architecture of the TCN module is illustrated in Figure 4.

At the input layer, the model constructs historical sequence features using a sliding time window mechanism. Each time step encapsulates diverse production-related dynamic information, including first order and second-order difference features, which are designed to capture the velocity and acceleration characteristics of production variations. For a historical window of length TTT, the input sequence can be formulated as:

(3) $$Xt=\{x_{t-T+1},x_{t-T+2},\ldots ,x_{t}\}$$

where $x_{t}$ denotes the multi-dimensional feature vector at time step *t*. By incorporating differential features, the model becomes more sensitive to the evolving trends in oil production, thereby enhancing its ability to detect subtle dynamic changes.

During the feature extraction stage, the TCN employs multi-layer dilated causal convolutions to model the input sequence. The causal convolutional structure ensures that the model exclusively utilizes current and historical information during prediction, thereby preventing information leakage from future time steps. Meanwhile, by progressively increasing the dilation rate across layers, the model substantially enlarges the temporal receptive field while maintaining relatively low computational complexity, enabling the capture of production patterns over extended temporal horizons. As illustrated, a three-layer convolutional architecture is adopted in this study, with dilation rates set to 1, 2, and 4, respectively, allowing the model to extract feature representations across multiple temporal scales. The general formulation of the dilated convolution operation can be expressed as:

(4) $$y_{t}=\sum_{i=0}^{k-1}w_{i}x_{t-d\cdot i}$$

where *K* denotes the kernel size, *d* represents the dilation rate, and $w_{i}$ corresponds to the convolutional kernel parameters.

To enhance training stability, layer normalization (LN) is incorporated after each convolutional layer to standardize feature distributions, while a Dropout mechanism is employed to mitigate the risk of overfitting [31]. Multi-scale temporal features extracted by dilated convolutional layers with varying dilation rates are subsequently integrated via feature concatenation, thereby yielding a more expressive and comprehensive temporal representation.

At the output stage, the fused features are fed into a fully connected layer, followed by a Dense layer to produce preliminary predictions of the logarithmic yield differences. In this study, a differencing-based prediction strategy is adopted to strengthen the model’s capacity to capture yield fluctuation dynamics, thereby improving both predictive stability and accuracy. These preliminary predictions serve as the baseline for the subsequent LightGBM-based residual learning subsystem, where they are further refined through a gradient boosting correction process, enabling precise compensation for temporal trend deviations.

#### 2.3.2. Residual Learning Based on LightGBM

**Figure 5:**
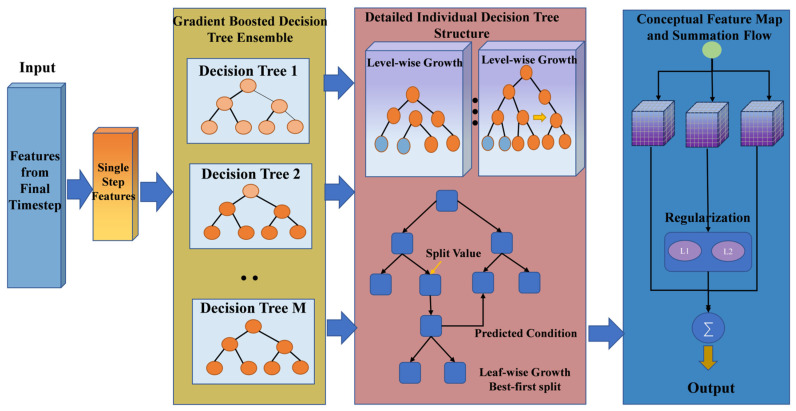
Schematic illustration of the LightGBM architecture and gradient boosting learning mechanism.

To enhance sensitivity to localized irregularities in sensor measurements, a residual correction module based on LightGBM is introduced. Rather than serving as an independent predictor, this module is specifically designed to model residual errors arising from sensor noise and nonlinear perturbations, as illustrated in Figure 5. These residuals often correspond to local production fluctuations or operational changes, such as choke valve adjustments, transient multiphase flow, or short-term injection/production variations, which are not fully captured by the TCN backbone.

To correct the prediction made at time step t, the LightGBM module takes as input a feature vector comprising four groups: (1) raw sensor measurements at time t, including choke size, wellhead pressure, and choke pressure differential; (2) rolling statistical features, namely 7-day and 30-day rolling means and standard deviations of daily oil production; (3) differential dynamic features, including first- and second-order differences of BORE_OIL_VOL at time t and their 7-day rolling means; and (4) the preliminary log-difference prediction $\Delta {\hat{y}}_{t}^{\left(0\right)}$ from the TCN backbone, which directly serves as the baseline for residual correction. Many of these features, notably the first- and second-order differences and rolling statistics, directly characterize transient behaviors and stability properties of well performance, offering a physical basis for interpreting the modeled residuals.

A gradient boosting ensemble is employed, in which successive decision trees are iteratively fitted to the residuals of the preceding model. During training, each new tree learns discriminative splits through optimal feature partitioning, and the outputs of all trees are aggregated under regularization to produce the final residual estimate. This iterative fitting process enables LightGBM to capture complex nonlinear relationships between the input features and prediction errors.

This residual-correction paradigm has been demonstrated to effectively enhance the accuracy of time-series forecasting [32]. Let the prediction of the base model at time step *t* be denoted as $\Delta {\hat{y}}_{t}^{\left(0\right)}$, and the corresponding ground-truth observation as ${\Delta y}_{t}$; the residual can thus be formulated as:

(5) $$r_{t}=\Delta y_{t}-\Delta {\hat{y}}_{t}^{\left(0\right)}$$

This residual encapsulates the variation patterns that remain unaccounted for by the base model. Analysis of feature importance from the LightGBM model can provide insight into which input variables most strongly contribute to these residuals, potentially revealing key drivers of localized flow instability or operational adjustments. LightGBM iteratively fits these residuals through an ensemble of decision trees, yielding an overall predictive function expressed as:

(6) $$F(x)=\sum_{m=1}^{M}f_{m}(x)$$

where $f_{m}(x)$ denotes the output of the *m* decision tree, and *M* represents the total number of trees. Through this iterative boosting process, the model is capable of capturing the intricate and nonlinear mapping between input features and prediction errors, thereby enabling targeted correction of the outputs produced by the temporal convolutional network (TCN) and substantially improving the overall modeling performance [33].

The module is trained in two phases. First, the TCN backbone is trained to predict the log-difference and its parameters are frozen. Second, the trained TCN generates predictions over the training set, and the residuals $r_{t}$ are paired with the feature vectors $x_{t}^{\mathrm{res}}$ to form a dataset on which LightGBM is fitted by gradient boosting with the mean squared error.

After training, the final production forecast for input window $X_{t}$ is obtained by:

(7) $$\Delta {\hat{y}}_{t}^{\left(0\right)}=f_{\mathrm{TCN}}(X_{t})$$

(8) $$\Delta {\hat{y}}_{t}^{\mathrm{final}}=\Delta {\hat{y}}_{t}^{\left(0\right)}+F(x_{t}^{\mathrm{res}})$$

(9) $${\hat{y}}_{t}^{\mathrm{final}}=y_{t-1}\times \exp(\Delta {\hat{y}}_{t}^{\mathrm{final}})$$

where $f_{\mathrm{TCN}}$ is the temporal convolutional network, F is the LightGBM model, and $y_{t-1}$ is the observed production at the preceding time step.

### 2.4. Adaptive Multi-Fidelity Bayesian Hyperparameter Optimization Strategy

Bayesian optimization (BO) efficiently optimizes expensive black-box functions by using a probabilistic surrogate model and an acquisition function to guide the search. It yields near-optimal solutions with few evaluations [34].

**Figure 6:**
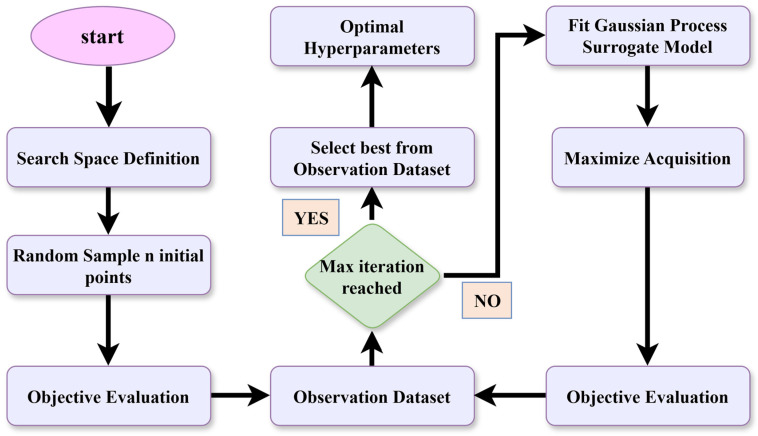
Workflow of Bayesian optimization for hyperparameter tuning.

Figure 6 illustrates the overall workflow of Bayesian optimization for hyperparameter tuning. At the initial stage of the optimization process, a set of candidate samples is randomly generated within the predefined parameter space, and the corresponding validation errors are obtained through model training (using time-series cross-validation on the training set). A surrogate model is then established to characterize the probabilistic distribution of the objective function, based on which the acquisition function is evaluated to determine the next set of hyperparameters for assessment. Through the continual refinement of observational data and iterative updating of the surrogate model, the search process progressively converges toward the region containing optimal hyperparameters.

Standard BO can still be costly when each model evaluation is expensive. To improve efficiency, we propose an adaptive multi-fidelity Bayesian optimization strategy with three components:

1. Adaptive search-space updating strategy. In the initial stage of optimization, a relatively large search domain is adopted to facilitate global exploration; as the number of iterations increases, the search space is progressively contracted around the neighborhood of the current optimum, thereby enabling a smooth transition from global exploration to localize fine-grained exploitation, and effectively eliminating redundant search regions.

2. Multi-fidelity evaluation mechanism. During the early phase of optimization (the first eight evaluations), a low-fidelity assessment is employed—where the Temporal Convolutional Network (TCN) is trained for a fixed number of 8 epochs—to enable rapid iterations and substantially reduce the computational cost per evaluation. Once the iteration count exceeds the fidelity-switching threshold (τ = 8), the procedure transitions to high-fidelity evaluation to obtain more stable and reliable estimates of model performance on the validation folds [35].

3. Dynamic surrogate updating. Throughout the optimization process, the surrogate model and the observed dataset are iteratively updated, allowing the search strategy to adaptively adjust the direction of parameter exploration and progressively converge toward the optimal hyperparameter configuration.

Upon completion of the optimization procedure, the resulting optimal hyperparameter configuration can be expressed as:

(10) $$x^{*}=\arg\min_{x}\;f(x)$$

where $X^{*}$ denotes the optimal parameter set identified via Bayesian optimization.

For clarity and completeness, Algorithm 1 presents the pseudocode of the proposed adaptive multi-fidelity Bayesian optimization approach. The algorithm delineates the full execution pipeline and explicates the implementation details of both the adaptive search-space adjustment and the multi-fidelity evaluation mechanism within the optimization process.
**Algorithm 1.** Adaptive Multi-Fidelity Bayesian Optimization.1: Input:2:    Hyperparameter search space H3:    Objective function f(x)4:    Maximum iterations T = 155:    Initial sample size N = 56:    Fidelity switching threshold τ = 87: Output:8:    Optimal hyperparameter x*9:    Initialize dataset D = ∅10: Randomly sample N configurations $\left\{x1,\ldots ,xN\right\}$ from H11: for i = 1 to N do 12:    Evaluate $f(x_{t})$ using low-fidelity training (8 epochs)13:    $D\leftarrow D\cup \{(x_{i},f(x_{i}))\}$
14: end for15: for t = N + 1 to T do 16:    Fit surrogate model p(f∣D)
17:    Select next candidate $x_{t}\leftarrow \arg\max\alpha (x|D)$
18:    if t≤τ then19:         Evaluate $f(x_{t})$ using low-fidelity training (8 epochs)20:    else21:         Evaluate $f(x_{t})$ using high-fidelity training22:    end if23:    Update dataset24:    $D\leftarrow D\cup \{(x_{t},f(x_{t}))\}$
25:    Adaptively shrink search space around the current best solution26: end for

## 3. Experimental Design

### 3.1. Workflow Overview

**Figure 7:**
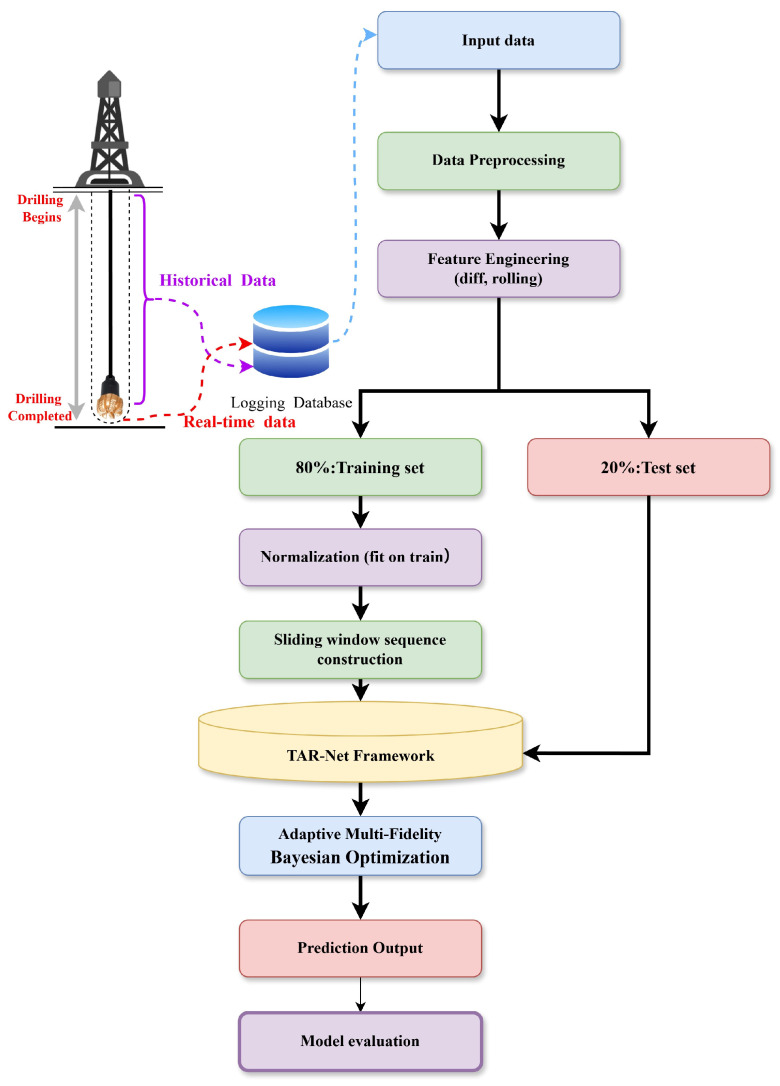
Overall framework of oil production forecasting based on the TAR-Net.

This study establishes an integrated experimental framework for the TAR-Net (Temporal Augmented Residual Network) model designed for petroleum production forecasting, as illustrated in Figure 7. Historical production data from oil wells, together with associated monitoring data, are first retrieved from the production database and subsequently fed into the model as inputs. The raw data are first cleaned by removing missing records, physically implausible values, and 3σ outliers, followed by feature engineering procedures including differencing and rolling statistical computations. The processed dataset is then partitioned into training and testing subsets in chronological order, with 80% of the samples allocated for model training and the remaining 20% reserved for model evaluation. To obtain reliable performance estimates for Bayesian hyperparameter optimization without touching the test set, we employed a time-series cross-validation strategy exclusively within the training set. Specifically, the training set was iteratively split into training folds and validation folds in a rolling-origin manner, preserving temporal order. The validation errors reported during optimization correspond to the average performance over these validation folds. The test set was strictly held out and used only for the final evaluation of the model with the selected hyperparameters. Data normalization is performed exclusively on the training set and subsequently applied to the testing set to ensure consistency.

Normalization is conducted using a standardization approach, defined as:

(11) $$x^{\prime }=\frac{x-\mu }{\sigma }$$

where x denotes the original data, μ represents the sample mean, and σ corresponds to the sample standard deviation.

**Figure 8:**
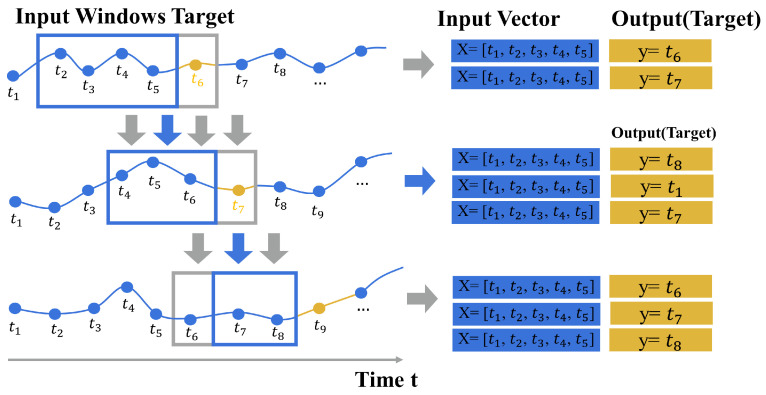
Schematic diagram of the sliding window mechanism.

Upon completion of data preprocessing, time-series samples are constructed using a sliding window mechanism, as depicted in Figure 8. This approach transforms continuous temporal sequences into fixed-length input segments while preserving the inherent temporal order. The sliding window advances along the time axis with a predefined length, thereby generating multiple samples. The resulting sequence samples serve as inputs to the model for subsequent training and prediction tasks.

During the model training phase, the processed time-series data are fed into the TAR-Net model for learning. The model leverages a temporal feature extraction architecture to capture dynamic patterns embedded within historical production data and to establish a mapping between input variables and petroleum output. Throughout the training process, the TCN (Temporal Convolutional Network) backbone employs the mean squared error (MSE) as the loss function, which is defined as:

(12) $$\mathrm{MSE}=\frac{1}{n}\sum_{i=1}^{n}{\left(y_{i}-{\hat{y}}_{i}\right)}^{2}$$

The LightGBM-based residual learning module is likewise trained via gradient boosting under the Mean Squared Error (MSE) loss, thereby ensuring full consistency between the residual compensation process and the objective function of the backbone model. Model parameters are optimized using the Adaptive Moment Estimation optimizer (Adam), which enhances both training efficiency and convergence stability. Upon completion of training, the model is applied to the test dataset to generate preliminary predictions of the logarithmic production differentials. These predictions are subsequently refined through residual learning, and the corrected differential outputs are transformed back into physical production values via an inverse exponential transformation, yielding the final oil production forecasts. The resulting predictions are then utilized to compute multiple evaluation metrics, thereby providing a comprehensive assessment of model performance. A fixed random seed (42) is adopted throughout the workflow to ensure experimental reproducibility.

**Figure 9:**
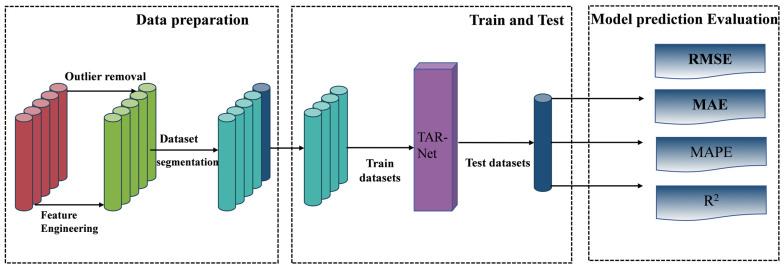
Experimental workflow for oil production time series forecasting using TAR-Net.

As illustrated in Figure 9, the aforementioned procedure can be further abstracted into three principal stages: data preparation, model training and testing, and prediction evaluation. Specifically, the data preparation stage constructs high-quality inputs through outlier treatment, feature engineering, and time-series segmentation. The model training and testing stage employs a sliding window mechanism to generate temporal samples, and leverages the Temporal Augmented Residual Network (TAR-Net) to achieve dynamic learning and forecasting. Finally, the prediction evaluation stage conducts a multi-metric, integrative analysis of model performance, thereby systematically validating the effectiveness and robustness of the proposed approach in oil well production time-series forecasting tasks.

### 3.2. Parameter Configuration

The search space for the Bayesian-optimized hyperparameters, along with the fixed parameters, is summarized in Table 2. The final optimal hyperparameter configurations obtained under different optimization strategies, including the proposed adaptive multi-fidelity Bayesian optimization, are presented below.

### 3.3. Evaluation Metrics

To evaluate the performance of the proposed model in the task of oil production time series forecasting, this study selects four commonly used metrics: Root Mean Square Error (RMSE), Mean Absolute Error (MAE), Mean Absolute Percentage Error (MAPE), and the coefficient of determination (R^2^). These metrics assess model performance from four perspectives: absolute error, relative error, goodness of fit, and stability on a logarithmic scale.

It is important to note that the MAPE metric becomes numerically unstable when the actual production value $y_{i}$ approaches zero, a common scenario during shut-in periods. To ensure a robust and meaningful calculation, data points where the actual daily oil production is strictly zero are excluded when computing the MAPE. This exclusion protocol is consistently applied across all models under comparison to maintain fairness.

The definitions of these metrics are as follows:

(13) $$\mathrm{RMSE}=\sqrt{\frac{1}{n}\sum_{i=1}^{n}{\left(y_{i}-{\hat{y}}_{i}\right)}^{2}}$$

(14) $$\mathrm{MAPE}=\frac{100\%}{n}\sum_{i=1}^{n}\left|\frac{y_{i}-{\hat{y}}_{i}}{y_{i}}\right|$$

(15) $$\mathrm{MAE}=\frac{1}{n}\sum_{i=1}^{n}|y_{i}-{\hat{y}}_{i}|$$

(16) $$R^{2}=1-\frac{\sum_{i=1}^{n}{\left(y_{i}-{\hat{y}}_{i}\right)}^{2}}{\sum_{i=1}^{n}{\left(y_{i}-\bar{y}\right)}^{2}}$$

In the above equations, n denotes the number of prediction points, $y_{i}$ denotes the actual value, and ${\hat{y}}_{i}$ denotes the predicted value.

## 4. Results and Discussion

We compare fourteen models for daily oil production forecasting: the proposed TAR-Net, its variants under different optimization strategies, and several representative baselines. In addition, a range of representative deep learning architectures and hybrid models are introduced as benchmark baselines. The objectives of this section are fourfold: (1) to substantiate the superiority of the proposed TAR-Net in predictive accuracy through a comprehensive comparison across multiple evaluation metrics; (2) to assess the effectiveness of Bayesian optimization (BO) in the hyperparameter tuning process via comparative analysis of models under different optimization strategies; (3) to further characterize the distributional properties and stability of prediction errors across models by examining the probability density distributions of residuals and the cumulative distribution of prediction error tolerance; (4) to investigate the physical interpretability of the LightGBM residual module by analyzing feature importance and linking key features to production phenomena; and (5) to illustrate the predictive advantages of the proposed model under complex production dynamics through comparative analysis of representative oil well production curves.

Table 3 presents a comparative evaluation of model performance in the time-series forecasting task of daily oil production. All models in Table 3 were evaluated under identical conditions: same data preprocessing, chronological 80/20 train/test split, sliding window of 30 days, and evaluation metrics. Hyperparameters of baselines were taken from their original publications or set to standard values, and the training protocol (optimizer, batch size, epochs) was unified across neural models. No extra tuning was performed for any baseline. Conventional recurrent neural network (RNN) and long short-term memory (LSTM) models exhibit relatively poor performance, with root mean square error (RMSE) values reaching 176.91 and 161.89, respectively, and mean absolute percentage error (MAPE) exceeding 12% in both cases, thereby revealing the intrinsic limitations of single recurrent architectures in capturing non-stationary fluctuations and multi-phase dynamics. Although the Transformer model reduces the RMSE to 152.54, its responsiveness to local abrupt variations remains inadequate. Hybrid architectures, such as CNN-LSTM and LSTM-Attention, achieve RMSE values of 145.64 and 138.92, respectively; while the incorporation of attention mechanisms yields marginal improvements, it fails to fundamentally resolve the coupled challenges of long-term dependency modeling and nonlinear feature representation. In contrast, the variants proposed in this study exhibit marked performance differentiation: the non-optimized TR-Net reduces the RMSE to 111.65, indicating that residual compensation mechanisms preliminarily enhance trend-tracking capability; the hybrid models LTR-Net and GTR-Net, integrating LSTM, GRU, and LightGBM, maintain stable coefficients of determination (R^2^) within the range of 0.9648–0.9663; whereas the purely temporal boosting decision tree model (TBL-Net) yields a substantially elevated RMSE of 225.16, underscoring its limited capacity to independently handle strongly non-stationary sequences. It should be noted, however, that these promising results are obtained on three wells from a single field (Volve). Direct generalization to other reservoir types or varying sensor quality environments remains to be validated, and the associated challenges are discussed later in this paper.

To address the structural limitations of the aforementioned models in jointly modeling temporal dependencies and nonlinear features, the proposed TAR-Net achieves optimal performance, with RMSE, MAE, and MAPE of 88.69, 38.56, and 7.8%, respectively, and an R^2^ of 0.9832. Compared with the LSTM and Transformer models, its RMSE is reduced by approximately 45.2% and 41.9%, respectively, demonstrating that the proposed model can more effectively characterize the complex dynamic variations in oil well production sequences.

**Figure 10:**
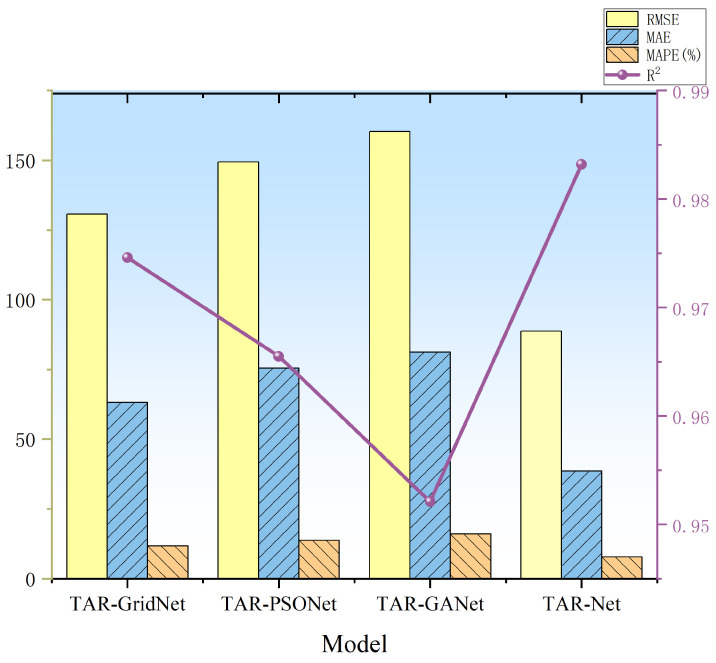
Predictive performance of TAR models under different hyperparameter optimization strategies.

Building upon the validated superiority of the overall model performance, we further investigate the influence of different hyperparameter optimization strategies on model efficacy. Table 4 summarizes the hyperparameter configurations obtained under various optimization schemes within the TAR-Net framework. Owing to its predetermined search trajectory, grid search is typically confined to selecting relatively optimal solutions from a limited set of parameter combinations. In contrast, population-based intelligent algorithms such as particle swarm optimization (PSO) and genetic algorithms (GA), despite offering a broader exploration space, are inherently susceptible to the initialization of populations and the stochasticity of iterative search paths, thereby impeding the formation of stable inter-module parameter synergies. Figure 10 presents a comparative analysis of the predictive performance achieved by models optimized via different strategies. From an overall perspective, substantial discrepancies are observed among the models derived from the three conventional optimization approaches, with PSO- and GA-optimized models exhibiting relatively larger prediction errors. In comparison, TAR-Net consistently attains superior performance across all evaluation metrics, reducing the root mean square error (RMSE) to 88.69, achieving a mean absolute percentage error (MAPE) of 7.8%, and improving the coefficient of determination (R^2^) to 0.9832. Collectively, these results indicate that multi-fidelity Bayesian optimization demonstrates enhanced search efficiency and robustness in high-dimensional spaces, enabling more effective coordination between the temporal convolutional network (TCN) and Light Gradient Boosting Machine (LightGBM) parameters, thereby substantially improving predictive accuracy.

**Figure 11:**
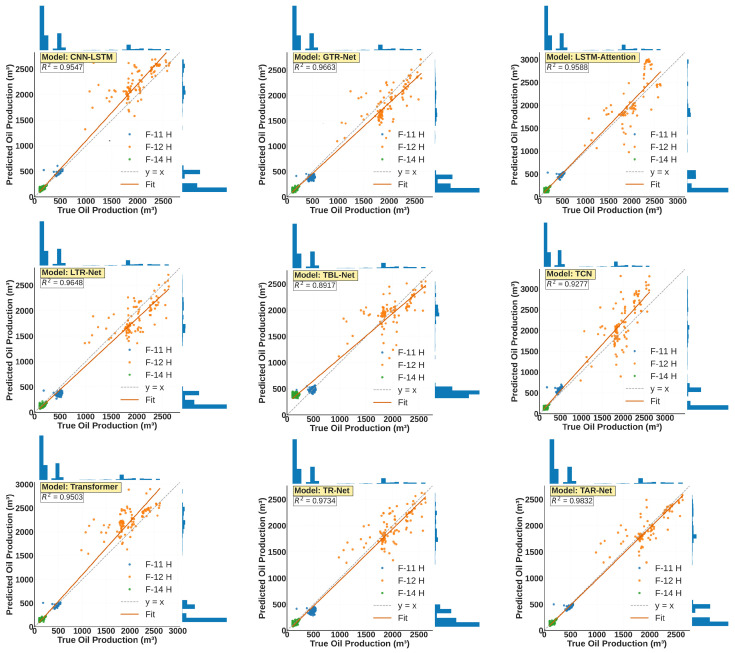
Comparison of scatter distributions and fitting performance for daily oil production prediction across different models.

Figure 11 illustrates the fitting performance of different models in the task of oil well production forecasting. Although base temporal models such as TCN and Transformer yield predictions that generally align with the diagonal distribution, noticeable scatter persists in the mid-to-high production ranges, indicating limited fitting stability. Models incorporating hybrid architectures achieve improved fitting accuracy, yet the predicted points still exhibit fluctuations across different production intervals. In contrast, the TAR series models demonstrate more concentrated fitting outcomes: TR-Net already shows a favorable fitting trend, while the proposed TAR-Net consistently aligns most closely with the ideal diagonal across all production intervals, accompanied by a marked reduction in scatter dispersion. It also attains the highest R^2^ value, accounting for the vast majority of variance in production fluctuations and sustaining strong fitting capability under complex dynamic conditions. The tighter clustering around the diagonal reflects reduced prediction variance and an improved bias–variance trade-off, further corroborating the stability of the proposed model.

**Figure 12:**
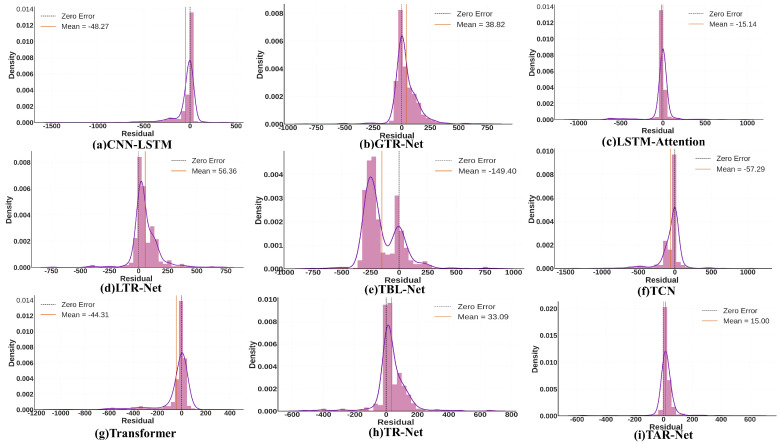
Comparative probability density distributions of prediction residuals for multiple models.

To further interrogate the distributional characteristics of prediction errors, a statistical analysis of model residuals is presented in Figure 12. Baseline deep learning models (Figure 12a,c,f,g) produce broad, skewed residual distributions, indicating systematic bias in capturing long-term dependencies. Ensemble models (Figure 12d,e) slightly narrow the error range but remain asymmetric or multimodal, suggesting that predictive stability remains susceptible to nonlinear fluctuations. Structural variant models (Figure 12b,h) progressively concentrate residuals toward the zero-error region, with improved overall symmetry, albeit with residual tailing effects still evident. In contrast, the Temporal Augmented Residual Network (TAR-Net) (Figure 12i) demonstrates the highest degree of concentration in its residual distribution, with the probability density curve forming a sharper and more symmetric peak around zero error. The mean residual is 15.00, representing the closest approximation to the zero-error baseline among all models. This characteristic underscores the model’s superior error concentration and its enhanced capability for suppressing extreme deviations. The narrower error distribution further confirms that TAR-Net effectively suppresses extreme prediction deviations, which is critical for reliable decision-making in engineering applications.

**Figure 13:**
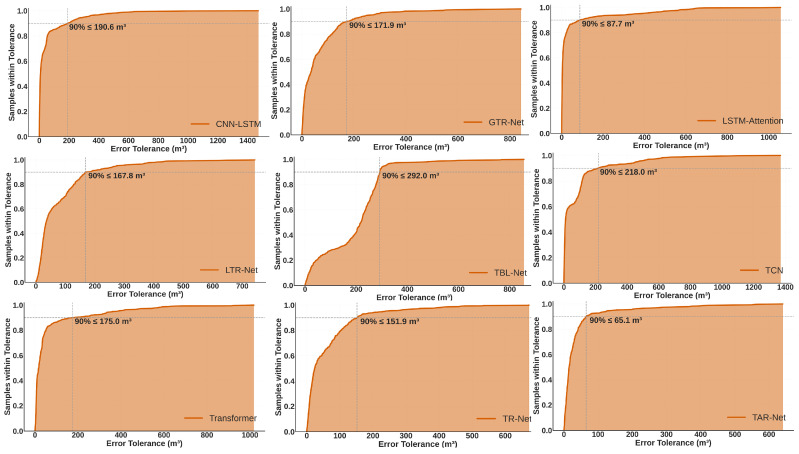
Cumulative error tolerance curves of absolute prediction errors for different evaluated models.

Figure 13 shows the cumulative distribution characteristics of prediction error tolerance for the evaluated models. Compared to alternative architectures, the cumulative trajectory of the Temporal Augmented Residual Network (TAR-Net) exhibits the most precipitous ascent within the low-error regime. Crucially, at a 90% sample coverage threshold, its error tolerance is restricted to 65.1 m^3^—a value substantially lower than those of the baseline models—thereby indicating an effective compression of the overall error magnitude. Morphologically, the TAR-Net profile displays a steep initial escalation before progressively plateauing, a dynamic that signifies the errors are predominantly constrained to the low-error domain, concomitant with a negligible prevalence of large-error samples. Conversely, the counterpart models sustain a protracted ascending phase across moderate-to-high error intervals, reflecting a markedly more dispersed error distribution. Ultimately, these findings corroborate the superior proficiency of TAR-Net in error regulation, endowing the predictive outcomes with enhanced congruity across the entire sample space.

**Figure 14:**
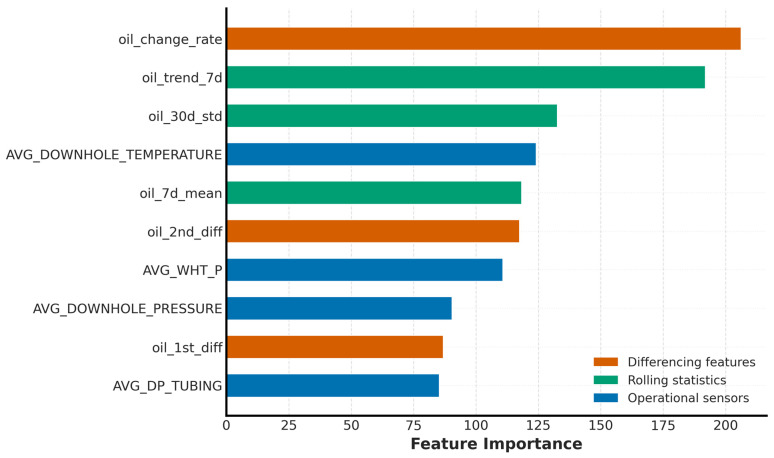
LightGBM feature importance for residual prediction.

Figure 14 displays the feature importance of the LightGBM residual module. Short-term differencing features, particularly oil_change_rate, dominate the ranking, indicating that the residual module primarily compensates for rapid, high-frequency production fluctuations—direct signatures of flow instabilities, transient slugging, or sudden operational adjustments—that the TCN backbone cannot fully capture. Rolling statistical features such as oil_trend_7d and oil_7d_mean contribute moderately by encoding local trend deviations, while operational variables including AVG_CHOKE_SIZE_P and AVG_WHT_P exhibit modest but non-negligible importance, confirming that choke valve adjustments and wellhead pressure variations partially drive residual behavior. This hierarchical structure validates the decoupled design of TAR-Net and establishes a transparent link between the residual correction mechanism and physically interpretable production phenomena.

**Figure 15:**
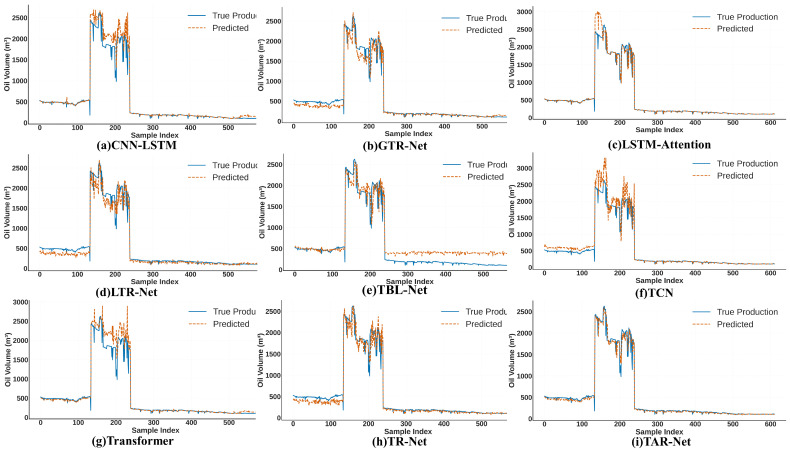
Comparison of the final predictive trajectories across distinct models.

A comprehensive comparison of the predictive trajectories yielded by the evaluated models across the complete time series is delineated in Figure 15. Macroscopically, while all assessed architectures successfully track the overarching trajectory of production variations, they exhibit pronounced discrepancies at distinct operational stages. During periods of relatively stable production, the predictive curves of the respective models demonstrate marginal divergence; however, under conditions of rapid production escalation and acute fluctuation, their responsive capacities bifurcate significantly. Specifically, the Convolutional Neural Network-Long Short-Term Memory (CNN-LSTM) model (Figure 15a) manifests amplitude deviations within highly volatile intervals, accompanied by temporal lags at certain peak loci. Concurrently, the Temporal Convolutional Network (TCN) (Figure 15f) exhibits markedly amplified predictive oscillations during high-production phases, thereby compromising local stability. Although the Transformer architecture (Figure 15g) preserves congruence with the macroscopic evolutionary trend, it persistently demonstrates offsets at multiple peak and trough coordinates. These observations underscore the inherent limitations of employing monolithic modeling mechanisms to concurrently resolve long-term trend evolutions and localized abrupt perturbations.

Structurally augmented models, conversely, attain a superior equilibrium between macroscopic trend fidelity and localized dynamic responsiveness. While the TR-Net (Figure 15h) closely approximates the empirical ground truth across the majority of temporal intervals, it still exhibits observable deviations within regimes of severe fluctuation. In stark contrast, the predictive trajectory of the Temporal Augmented Residual Network (TAR-Net) (Figure 15i) maintains an exceptional degree of consistency spanning the entire temporal continuum. It not only responds instantaneously to transitional dynamics in the vicinities of peaks and troughs but also sustains robust stability during the subsequent phase of rapid production attenuation. Notably, its corresponding predictive curve is devoid of any conspicuous amplitude amplification or temporal hysteresis. Comprehensively, the strategic integration of temporal augmentation and residual compensation mechanisms enables the model to achieve an optimal synergy between macroscopic trend tracking and microscopic fluctuation delineation, thereby ensuring robust and continuous predictive efficacy under highly complex production dynamics.

In addition to predictive accuracy, practical deployment in field monitoring systems imposes stringent requirements on computational efficiency and scalability. To systematically quantify these aspects, Table 5 compares the training time, inference latency, asymptotic time complexity, memory footprint, and real-time suitability of all 16 evaluated models. All measurements were performed on the same hardware platform (Intel Xeon CPU, NVIDIA RTX 3090 GPU) with identical software stacks, ensuring fair and reproducible comparisons.

Several important trends emerge from Table 5. The proposed TAR-Net achieves a training time of 360 s, which is substantially lower than that of Transformer (1520 s) and CNN-LSTM (1210 s), while maintaining an inference latency of only 0.8 ms per sample. This sub-millisecond inference is orders of magnitude smaller than the typical sampling interval of oilfield sensors, confirming its suitability for near-real-time production monitoring and online decision-making. From a complexity perspective, the TCN backbone contributes a linear O(T × K × F) term, and the LightGBM residual module adds O(N log N), effectively avoiding the O(T^2^) bottleneck that penalizes self-attention-based models such as Transformer and LSTM-Attention. These quadratic dependencies directly translate into the elevated training and inference costs observed for those architectures. Although the purely tree-based TBL-Net achieves the best efficiency (90 s training, 0.3 ms inference), this comes at the expense of severely degraded predictive accuracy. In contrast, TAR-Net and its optimized variants (TAR-GridNet, TAR-PSONet, TAR-GANet) occupy a uniquely favorable position in the accuracy-efficiency trade-off, combining state-of-the-art prediction performance with modest memory usage (~150 MB) and low latency. These characteristics make TAR-Net particularly attractive for deployment on edge computing devices commonly found in production field stations, where both predictive fidelity and computational resource constraints must be satisfied simultaneously.

## 5. Conclusions

### 5.1. Summary

In this work, we propose a Temporal Augmented Residual Network (TAR-Net) for oil well production forecasting. The proposed framework follows a decoupled modeling strategy that explicitly separates long-term trend modeling from residual error correction. A multi-scale dilated temporal convolutional network (TCN) is employed as the backbone to capture the overall temporal evolution of production data, while a LightGBM-based residual correction module is introduced to model the localized fluctuations and nonlinear patterns not fully captured by the backbone. This design enables more effective handling of both global trends and local variations.

For feature engineering, a hierarchical temporal representation is constructed using rolling statistics and differencing operations. This approach allows the model to capture local statistical characteristics, such as mean levels and fluctuation patterns, while preserving the underlying temporal dynamics of the production process. In addition, an adaptive multi-fidelity Bayesian optimization strategy is adopted for hyperparameter tuning. By progressively refining the search space and leveraging multi-fidelity evaluations, the optimization process is made more efficient while maintaining stable model performance across different conditions.

Beyond predictive performance, the proposed framework provides a clear and interpretable structure by separating trend modeling from residual correction. While the two components are not strictly independent, this design enables effective specialization of sub-tasks and contributes to improved forecasting accuracy. The proposed method offers a practical solution for oil well production prediction and provides a basis for future work involving the integration of physical knowledge and multi-source data. Furthermore, feature importance analysis of the LightGBM residual module reveals that short-term differencing features and operational sensor variables—such as choke size and wellhead pressure—dominate the residual correction process. This indicates that the residual module not only improves predictive accuracy but also provides physically interpretable insights into production dynamics, including flow instabilities, transient operational adjustments, and localized fluctuations. The explicit separation of trend modeling and fluctuation compensation thus enhances both forecasting performance and model transparency. The results suggest that explicitly separating temporal dependency modeling from residual correction provides a practical approach for handling complex sensor-derived time series in industrial applications.

It should be noted, however, that the reported results are obtained on three wells from a single field (Volve). Direct generalization to other reservoir types or varying sensor quality environments remains to be validated. The decoupled architecture of TAR-Net facilitates modular adaptation, allowing the temporal backbone and residual correction module to be fine-tuned for new fields. Preliminary analyses indicate that the model maintains stable performance under moderate sensor noise or partial missing data, although extreme conditions may still affect predictions. Future work will focus on cross-field transfer learning, anomaly-aware modeling, and integration of multi-source data to enhance generalization and robustness across diverse reservoir and sensor conditions.

### 5.2. Limitations and Future Work

It is worth noting that real-world sensor data inherently contain noise and measurement uncertainties. Therefore, the evaluation conducted on raw field data implicitly reflects the robustness of the proposed model under realistic sensor conditions. Although this study has achieved notable advances, several limitations persist:

(1) Limited cross-field generalization validation: The current experimental validation is exclusively conducted on three wells from the Volve field. While the results are promising, it is important to acknowledge that geological characteristics and development regimes vary significantly across different oilfields. Consequently, the model’s transferability to other fields has not been empirically verified. The decoupled architecture may require re-optimization or fine-tuning for data distributions that differ substantially from the North Sea reservoir conditions represented by the Volve dataset.
(2) Sensitivity to sensor quality and noise characteristics: The model’s performance hinges on the availability of ample, high-fidelity historical records. In real-world scenarios, sensor measurements inevitably suffer from varying degrees of noise, drift, or even missing values due to harsh operating conditions. Our framework’s robustness has not been rigorously validated under regimes of data scarcity or elevated, non-stationary sensor noise. Specifically, the differencing features and the LightGBM residual module, while sensitive to local fluctuations, might inadvertently amplify high-frequency noise in low signal-to-noise ratio environments.
(3) Insufficient physical mechanistic grounding: The present framework is entirely data-driven, and the residual compensation process lacks explicit anchoring in reservoir engineering principles, thereby constraining interpretability and reliability under extreme operating conditions. Nevertheless, the feature importance analysis presented in Section 4 provides a first step toward linking the residual corrections to physically meaningful production events, demonstrating that data-driven interpretability can partially mitigate this limitation.

Building upon the identified constraints, future research could advance along the following trajectories:

1. Cross-field transfer validation: Systematically evaluate TAR-Net on diverse public datasets and explore domain adaptation techniques to enhance cross-reservoir generalization.

2. Sensor-robust extensions: Develop noise-aware training strategies and adaptive feature selection mechanisms to maintain prediction stability under varying sensor fidelity and missing data scenarios.

3. Physics-informed integration: Incorporate reservoir engineering constraints into the learning framework to improve interpretability and reliability under extreme conditions.

4. Computational efficiency for deployment: Refine model architecture and training protocols to curtail computational demands and accelerate real-time engineering deployment.

## Figures and Tables

**Table 1:** Statistics of Training and Test Samples Per Well.

Well Name	Training Set Samples	Test Set Samples	Total
F-11H	872	218	1090
F-12H	717	179	896
F-14H	2141	535	2676
Total	3730	932	4662

**Table 2:** Hyperparameter Search Space for the TAR-Net Model.

Hyperparameter	Category	Search Range/Fixed Value
Seed	Fixed Parameters	42
Time_Steps	Fixed Parameters	30
Batch_Size	Fixed Parameters	64
Epochs	Fixed Parameters	100
Reg_Alpha/Reg_Lambda	Fixed Parameters	0.1/0.5
Min_Child_Samples	Fixed Parameters	30
TCN_Filters	Bayesian Optimization	64–256
Kernel_Size	Bayesian Optimization	2–7
Dropout_Rate	Bayesian Optimization	0.2–0.5
LR	Bayesian Optimization	1 × 10^−5^–1 × 10^−2^
LGBM Estimators	Bayesian Optimization	200–800
LGBM Learning Rate	Bayesian Optimization	0.01–0.2

**Table 3:** Comparison of time series forecasting performance for oil production across different models.

Model	RMSE	MAE	MAPE	R^2^
TR-Net	111.65	63.47	12.93%	0.9734
LTR-Net	128.39	79.90	17.78%	0.9648
GTR-Net	125.64	70.07	15.12%	0.9663
TBL-Net	225.16	189.47	36.26%	0.8917
TCN	184.01	85.63	27.53%	0.9277
CNN-LSTM	145.64	58.31	11.90%	0.9547
LSTM-Attention	138.92	45.02	8.32%	0.9588
Transformer	152.54	64.36	11.93%	0.9503
LSTM	161.89	75.69	12.95%	0.9377
RNN	176.91	83.12	15.64%	0.9300
TAR Net w/o Diff	98.42	42.10	11.4%	0.9621
TAR Net w/o Stat	105.78	49.02	10.8%	0.9590
TAR-GridNet	130.66	63.17	11.71%	0.9746
TAR-PSONet	149.36	75.49	13.71%	0.9655
TAR-GANet	160.31	81.26	15.97%	0.9521
TAR-Net	88.69	38.56	7.8%	0.9832

**Table 4:** Optimal hyperparameter configurations obtained using different optimization strategies.

Model	TCN Layers	TCN Filters	Kernel Size	Dropout	Learning Rate	LGBM Estimators	LGBM Learning Rate
TAR-GridNet	3	128	3	0.35	0.0010	500	0.08
TAR-PSONet	3	192	4	0.30	0.0008	650	0.06
TAR-GANet	3	160	5	0.28	0.0012	540	0.07
TAR-Net	3	224	4	0.26	0.0006	720	0.05

**Table 5:** Computational Cost and Scalability Comparison Across All Evaluated Models.

Model	Training Time (s)	Inference Time per Sample (ms)	Complexity	Memory Usage (MB)	Suitability for Real-Time
RNN	850	2.6	O(T × H^2^)	85	Fair
LSTM	980	2.8	O(T × H^2^)	120	Fair
TCN	480	1.1	O(T × K × F)	160	Good
Transformer	1520	5.2	O(T^2^ × d_model_)	450	Poor
CNN-LSTM	1210	4.1	O(T × (K × F^2^ + H^2^))	280	Fair
LSTM-Attention	1050	4.5	O(T^2^ × H)	250	Fair
TBL-Net	90	0.3	O(N log N)	50	Excellent
LTR-Net	550	1.4	O(T × H^2^ + N log N)	135	Good
GTR-Net	520	1.5	O(T × H^2^ + N log N)	140	Good
TR-Net	445	0.9	O(T × K × F + N log N)	155	Good
TAR Net w/o Diff	375	0.8	O(T × K × F + N log N)	150	Good
TAR Net w/o Stat	370	0.8	O(T × K × F + N log N)	150	Good
TAR-GridNet	410	0.8	O(T × K × F + N log N)	150	Good
TAR-PSONet	380	0.8	O(T × K × F + N log N)	150	Good
TAR-GANet	395	0.8	O(T × K × F + N log N)	150	Good
TAR-Net	360	0.8	O(T × K × F + N log N)	150	Good

## Data Availability

The raw data used in this study are publicly available in the following data repository: GitHub-awgeo/Volve_field_data: Exploring various geoscience Python libraries (including Lasio, Welly and Striplog) with the Volve Field data set.

## Abbreviations

TAR-Net	Temporal Augmented Residual Network
TR-Net	TAR-Net without optimization
LTR-Net	LSTM-LightGBM Hybrid Net
GTR-Net	GRU-LightGBM Hybrid Net
TBL-Net	Temporal Boosting LightGBM Network
TCN	Temporal Convolutional Network
CNN-LSTM	Convolutional Neural Network–Long Short-Term Memory hybrid model
LSTM-Attention	Long Short-Term Memory with Attention mechanism
Transformer	Transformer Network
LSTM	Long Short-Term Memory
RNN	Recurrent Neural Network
TAR-GridNet	TAR-Net with Grid Search Optimization
TAR-PSONet	TAR-Net with Particle Swarm Optimization
TAR-GANet	TAR-Net with Genetic Algorithm Optimization
BO	Bayesian Optimization
GRU	Gated Recurrent Unit
SVR	Support Vector Regression
RF	Random Forest
LightGBM	Light Gradient Boosting Machine
TAR-Net w/o Diff	TAR-Net without Difference Features
TAR-Net w/o Stat	TAR-Net without Statistical Features
